# The Influence of Sleep Talking on Nocturnal Sleep and Sleep-Dependent Cognitive Processes

**DOI:** 10.3390/jcm11216489

**Published:** 2022-11-01

**Authors:** Milena Camaioni, Serena Scarpelli, Valentina Alfonsi, Maurizio Gorgoni, Mina De Bartolo, Rossana Calzolari, Luigi De Gennaro

**Affiliations:** 1 Department of Psychology, Sapienza University of Rome, Via dei Marsi 78, 00185 Rome, Italy; 2 Body and Action Lab, IRCCS Fondazione Santa Lucia, Via Ardeatina 306, 00179 Rome, Italy; 3 Department of General Psychology, University of Padova, Via Venezia 8, 35131 Padova, Italy

**Keywords:** sleep talking, somniloquy, sleep pattern, dreams, memory consolidation, parasomnia

## Abstract

Background: Sleep talking (ST) is characterized by the production of unaware verbal vocal activations (VBs) during sleep. ST seems potentially linked to linguistic and memory consolidation processes. However, sleep and dream characteristics and the relationship between verbal vocalizations (VBs) and cognitive functions are still unknown. Our study aimed to investigate qualitative sleep and dream features in sleep talkers (STs) compared to healthy subjects (CNTs) through retrospective and longitudinal measures and explore the relationship between ST and memory consolidation. Methods: We recruited N = 29 STs and N = 30 CNTs (age range of 18–35). Participants recorded their dreams and filled out sleep logs for seven consecutive days. Vocal activations of STs were audio-recorded. On the eighth day, we administered a word-pair task. Results: We showed that STs had significantly worse self-reported sleep quality. VBs were positively correlated with sleep fragmentation and negatively associated with the oneiric emotional load. No difference between groups was found in the memory consolidation rate. Conclusions: Although ST is a benign phenomenon, we revealed that ST is associated with more sleep alterations and lower emotional intensity of dreams. In this vein, we support that ST depends on sleep fragmentation and could represent a potential window into sleep-dependent cognitive processes.

## 1. Introduction

Somniloquy (Sleep Talking -ST) is defined as the production of unaware linguistic vocalizations during sleep [1], to be differentiated from other utterances such as mumbling, laughing, groaning, and whistling [2,3]. Other utterances, defined as Non-Verbal episodes [2,4], and ST could often occur together, as shown in a non-comorbid ST sample [2].

ST is classified as “other symptoms and normal variants” of the parasomnias and is considered a benign phenomenon [5]. However, ST episodes would appear to affect nighttime sleep. A recent laboratory study showed that sleep-talking subjects have lower sleep efficiency than healthy subjects [2].

One of the most interesting implications is the possibility of considering ST as a phenomenon that would allow direct access to the mental activity occurring during sleep [4]. Accordingly, the literature defines ST as Dream-enactment behavior (DEB) [1,4] due to the reported parallelism between ST and dream content [6,7,8]. Some authors identified a high degree of concordance between words pronounced in ST episodes and dream reports [6], suggesting that ST may reflect cognitive processes. Specific EEG patterns preceding the onset of ST episodes mirror the EEG topography of wake linguistic planning and production [2]. These results suggested an elaboration of language occurring during sleep [1,2,4].

Furthermore, some studies on parasomnias revealed task-related information in their parasomniac episodes [9,10,11]. Specifically, Uguccioni et al. [11] assessed verbal declarative memory consolidation and observed a replay of material learned during an ST episode produced by a patient with REM Behavior Disorder (RBD). These results suggest that motor activations during parasomnia events help cognitive processing during sleep by an overt replay, promoting sleep-dependent learning.

The available literature supported the idea that ST would allow direct access to sleep mentation or direct observation of cognitive processes that occur during sleep [1,2,4,11].

Although ST is one of the most common sleep behaviors in the general population [12], the knowledge about this phenomenon is still poor. No studies have investigated sleep talkers’ sleep and dream characteristics longitudinally. Therefore, we have investigated qualitative sleep and dream characteristics in sleep talkers compared to healthy subjects through retrospective and longitudinal measures. We also evaluated whether any influence on sleep was due strictly to the ST or whether other utterances (Non-verbal STs-NVBs) could impact too.

Moreover, the literature on the relationship between ST and memory consolidation is scarce. Consequently, we carried out an exploratory study on the potential impact of ST on memory consolidation in sleep talkers without other comorbidities. Accordingly, we tested two alternative hypotheses: (A) The replay of verbal content on STs increases the sleep-dependent gain (defined as the difference between morning and evening recall) in the ST group, or (B) the sleep fragmentation due to STs is associated with a decreased memory performance.

## 2. Materials and Methods

### 2.1. Participants

**Figure 1:**
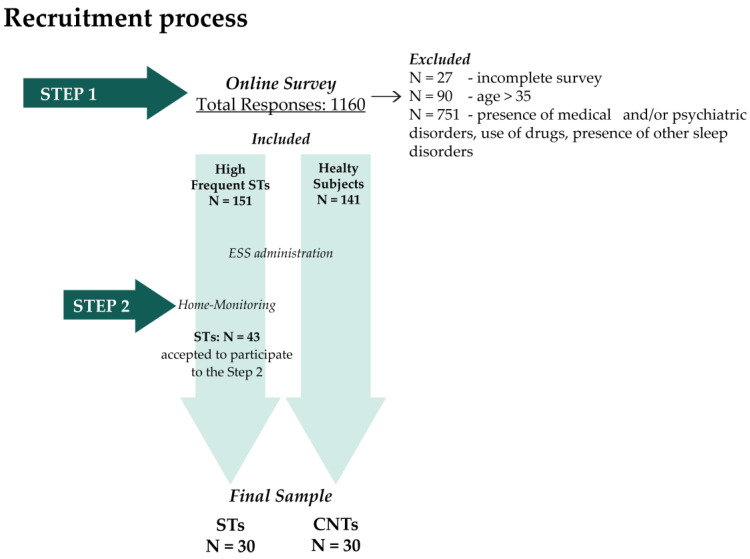
Recruitment process. Abbreviations: STs, Sleep Talkers’ group; CNTs, healthy; ESS, Epworth Sleepiness Scale.

We conducted a two-step recruitment process (Figure 1): An online survey and home monitoring specific for STs (see Mangiaruga et al. [2]).

The first step consisted of an online survey administered to the general population (age range 18–75 y.o.) through the most popular digital platforms (i.e., Facebook, Instagram, Whatsapp). The survey included the provision of consent, an ad hoc questionnaire assessing general health (Table S1) [2], the Pittsburgh Sleep Quality Index (PSQI) [13], and the Munich Parasomnia Questionnaire (MUPS) [14]. STs and CNTs groups were required to meet the following inclusion criteria: Age range: 18–35 y.o; no sleep disorders; no neurological, psychiatric, and medical conditions; no medications; no drug or alcohol abuse. ST subjects had frequently experienced ST episodes during sleep, reporting in the MUPS a score of 5–7 on the item related to ST episodes. In addition, for the control group, we ensured that there was no presence of other sleep utterances through the specific MUPS item (Do you sigh or moan loudly or continuously during sleep?).

Eligible participants (STs and CNTs) also filled out the Epworth Sleepiness Scale (EES) [15]. Before the ESS distribution and the STs’ participation in the second step, we collected informed consent.

Within 1160 responses, N = 27 were excluded because they did not complete the survey and N = 90 individuals were older than 35 years old. Among the remaining 1043 young adults, N = 151 declared themselves highly frequent STs and fulfilled the other inclusion criteria. Within this sample, N = 43 STs consented to participate in the second recruitment step. 

The second step consisted of home monitoring for one week to verify the presence and frequency of ST episodes. The STs were instructed to use an open-source voice-activated recording app installed on their personal smartphones during sleep to capture ST episodes.

As a final criterion, we selected STs (N = 30) who produced at least one ST episode during home monitoring. From a sample of N = 141 eligible healthy subjects, we selected N = 30 CNTs in the final sample balanced by gender and age (see Figure 1).

All participants signed informed consent before participating in the study. The present study complied with the Declaration of Helsinki and was approved by the Institutional Review Board of the Department of Psychology of the Sapienza University of Rome (protocol number: 0000226).

### 2.2. Procedures

The experimental procedure lasted 8 days. We required the ST and CNT groups to audio-record their dreams and fill out sleep diaries through an online-portal [16] within 15 min after last waking up every morning for seven days.

The STs group also used the voice-activated recording app during sleep to capture ST episodes. We chose audio-recording of dream reports to ensure high compliance and more accurate reports of mental activity [17]. Moreover, we trained all participants to record any remembered mental activity as accurately as possible and distinguish reports when they recalled more than one.

On the eighth day, we administered a declarative memory task (Word pair-associated learning task—WPT) remotely. The memory task consisted of three phases: The learning phase, immediate recall (evening), and delayed recall (morning). Each participant was tested on separate days according to the following timeline: (a) We required the subject to log on to Google meet at 9:30 p.m. to perform the learning phase and then the immediate recall. An experimenter ensured that the room was as quiet as possible and that there were no distracting external stimuli; (b) after the WPT administration, we instructed the subject to go to bed at 11:00 p.m. and wake up at 7:00 a.m., for a total of 8 h of bedtime; (c) the next morning, the participant filled out sleep diaries. STs audio-recorded their vocal activations during sleep; (d) at 8:00 a.m., the subject logged on to Google meet to perform the delayed recall. We ensured performance of the delayed recall occurred 45 min after waking up to avoid the effects of sleep inertia on performance [18,19].

### 2.3. Measures

#### 2.3.1. Pittsburgh Sleep Quality Index (PSQI)-Italian Validation

PSQI is a retrospective self-report questionnaire administered to assess sleep quality over the last month. The questionnaire consists of 19 items that generate seven variables: Subjective sleep quality, sleep latency, sleep duration, habitual sleep efficiency, sleep disturbance, use of sleep treatment, and the presence of diurnal dysfunction. These components produce a global score ranging from 0 to 21. A global score greater than 5 indicates the presence of a sleep disorder.

#### 2.3.2. Epworth Sleepiness Scale (ESS)-Italian Validation

ESS is a questionnaire that assesses daytime sleepiness. The questionnaire requires one to indicate the chance of falling asleep in the reported situations on a scale of 0 to 3. A total score greater than 10 indicates abnormal daytime sleepiness.

#### 2.3.3. Sleep Diaries

The sleep diaries acquired the subjective assessment of night sleep. The following information was collected: Bedtime and light off time, the amount of time (in minutes) of falling asleep after light off (sleep onset latency—SOL), subjective duration in minutes (intra-sleep wakefulness—ISW), subjective total sleep duration, time of final awakening, and rating on a 5-point Likert scale of sleep quality (3 items: Depth (from 1 very light sleep to 5 very depth sleep), Quiet (from 1 very disturbed sleep to 5 very quiet sleep), and Restless (from 1 very low rest sleep to 5 very high rest sleep)). The variables rated on the Likert scale represent a direct judgment of the subject’s sleep quality.

Then, from the raw data, other variables were extracted:

- Total Sleep Time in minutes (TST), computing the amount of time spent asleep.
- Total Bed Time (TBT), corresponding to the amount of time from the light off and the final awakening).
- Sleep Efficiency (SE = TST/TBT × 100).

#### 2.3.4. Dream Reports

Two independent judges (RC and MB) consistently evaluated each group’s dream reports. After removing all repetition and subjects’ inferences (pruning), the researchers reported the total number of words obtained, defined as the Total Words Count (TWC).

Moreover, judges rated emotional intensity (EL), vividness (VV), and bizarreness (B) on a Likert scale from 1 to 6 points [20]. The inter-rater reliability for each scale was substantial (Cohen’s K > 70). Differences between the two judges were consensually solved.

The VV variable was evaluated according to the following criteria: (1) No image, only thinking of objects; (2) very vague; (3) less vague; (4) moderately clear and vivid; (5) clear and reasonably vivid; (6) clear and vivid as normal vision. 

Regarding the B variable, the judges assigned the score considering bizarre elements (objects, characters, actions, or roles improbable or impossible) and bizarre script (physically/logically/ discontinuity improbable or impossible, improbable, or impossible settings).

Moreover, the judges counted the words separately with negative (Nw) and positive (Pw) emotional load. The two judges consensually solved any discordance. Table S2 reported an example of content dream evaluation.

#### 2.3.5. Vocal Activations

ST episodes were evaluated by two experimenters (RC and MDB) by listening and transcribing the voice recordings. The vocal activations were classified into ST Verbal (VBs—intelligible and non-intelligible) and Non-verbal ST (moaning, crying, laughter, and long sighs).

#### 2.3.6. Word-Pair Task

WPT was used to assess verbal declarative memory. We administered auditory stimuli to involve the subject’s phono-articulatory production and ensure the correct understanding of stimuli. The task included 50 pairs of semantically related words. In addition, 4 dummy pairs of words—excluded from further analyses—were presented at the beginning and end of the list to avoid primacy and recency effects. From an Italian database of nouns (“Lexvar”) [21], we selected words characterized by two or three syllables, high imaginability, concreteness, and familiarity, and a low emotional connotation. We presented the pair list with a 1-sec intra-stimulus time interval (duration between two paired words) and a 5-sec inter-stimulus (duration between one pair and the following) time interval. During the learning phase, participants repeated each pair immediately after its presentation. After learning, the immediate recall session followed, in which only the first word of each pair (cue word) was administered. Participants had to name the second word aloud in this phase with unlimited time. The experimenter gave the participants feedback of “yes” for correct answers and “no” for wrong answers. We presented the list repeatedly, three times at most (number of trials), until participants reached at least 60 percent of the word pairs. We administered all WPT lists randomly. The consolidation rate (Gain) was computed as the difference between correct words retrieved during the last delay and immediate recall.

### 2.4. Statistical Analyses

The statistical procedures were carried out using the Statistical Package for Social Sciences (SPSS, Inc., Chicago, IL, USA) version 25.0. We performed the appropriate parametric or non-parametric analysis depending on the data distribution.

#### 2.4.1. Demographic Characteristics

We compared age (Mann–Whitney U test) and gender (Chi-square) between the two groups to check the lack of significant differences.

#### 2.4.2. Qualitative Characteristics of Sleep and Dreams in STs

We compared retrospective and longitudinal measures between STs and CNTs to assess any differences in sleep variables. We performed the Mann–Whitney U test to compare the PSQI global score of the two groups and the unpaired Student t-test for the ESS score. Then, we compared the week’s mean of each sleep and dream variable between the two groups. The appropriate comparisons (Mann–Whitney U test or unpaired Student t-test) were performed. The significance level was corrected for multiple comparisons using the Benjamini–Hochberg False Discovery Rate (FDR) multiple testing correction [22].

In order to verify the relation between ST and sleep and mental activity, we correlated the means of vocal activations (VBs and NVBs) with the means of sleep and dream variables during the experimental week. We performed Pearson or Spearman correlations according to the variables’ distribution (Table S3).

#### 2.4.3. Memory Performance

We compared the two groups’ Gain scores (unpaired Student t-test) to assess memory performance. Moreover, we performed an unpaired Student t-test to compare the number of trials and the number of correct words recalled in the evening, to verify no differences between STs and CNTs in the initial encoding (baseline). 

Specifically, we correlated vocal activations recorded during the WP night by the STs group with gain, aiming to investigate ST’s positive or negative influence on sleep-dependent memory consolidation.

## 3. Results

We recruited sixty participants: N = 30 subjects with frequent episodes of ST (STs) and N = 30 healthy individuals (CNTs), balanced by gender and age (range 18–35 years). However, N = 1 ST was excluded from all analyses due to technical problems with the voice-activated recording app during the experimental week. Therefore, the final sample included N = 29 STs (F = 23; mean age: 23.48 sd: ±2.81 (se): ±0.52) and N = 30 CNTs (F = 24; mean age: 24.27 sd: ±3.24; (se): ±0.59). There were no significant differences between the two groups for age and gender (Table 1).

STs produced a total of 259 VBs (week mean (sd): 1.32 (1.08)) and 1377 NVBs (week mean (sd): 7.04 (6.03)).

Moreover, a second ST subject was excluded from the analysis of memory performance and sleep variables of the eighth night due to an audio technical problem with the administration of the WPT lists during the learning phase. According to this specific factor, the exclusion of this participant only affects the results reported in Table 4, Table S4 and Table S5.

### 3.1. Qualitative Characteristics of Sleep and Dreams in STs

Comparing PSQI and ESS scores showed that STs had significantly lower subjective sleep quality (U = 633.50; adj-*p* = 0.032) than CNTs, while there were no differences in perceived sleepiness (t = −2.13; *p* = 0.202). Moreover, the two groups did not report significant differences in sleep variables (Table 2).

**Figure 2:**
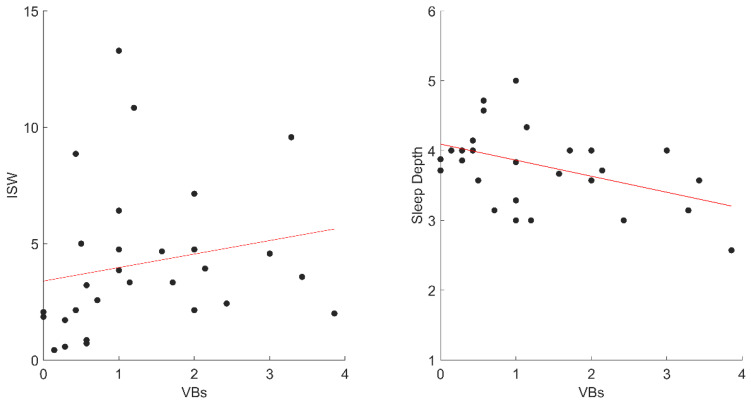
The significant correlations between VBs and sleep diaries variables in STs. Abbreviation: VBs, ST Verbal; ISW, Intra-Sleep Wakefulness.

Furthermore, the correlations between vocal activations and sleep diary variables showed a positive relation between VBs and ISW (r_ho_ = 0.42; *p* = 0.023) and a negative correlation between VBs and Depth (r_p_ = −0.44; *p* = 0.017) (Figure 2). Conversely, NVB activations did not significantly correlate with any sleep variables.

Regarding dream variables, no significant differences were observed between STs (N = 28) and CNTs (N = 24) who reported oneiric contents during the experimental week (Table 3).

**Figure 3:**
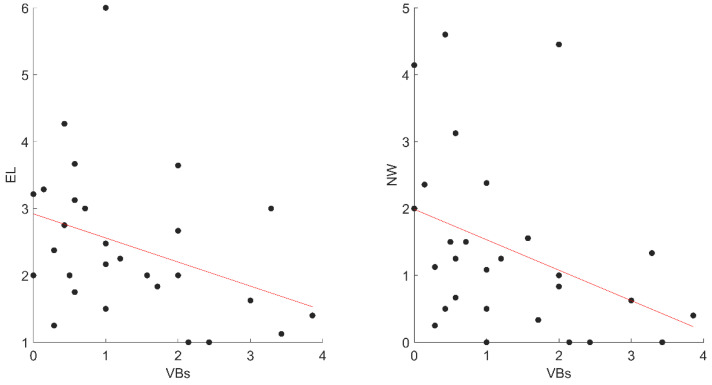
The significant correlations between VBs and dreams variables in STs. Abbreviations: VBs, ST Verbal; EL, Emotional Intensity; NW, negative words, STs, Sleep Talkers’ group.

However, we found that VB activations negatively correlated with EL (r_ho_ = −0.42; *p* = 0.028) and Nw (r_ho_ = −0.46; *p* = 0.015) (Figure 3).

### 3.2. Memory Performance

During the eighth night, STs produced a total of 18 VBs (mean (sd): 0.67, 0.88) and 154 NVBs (mean (sd): 5.70, 6.64).

We found no differences between the two groups concerning the number of trials (t = −0.65, *p* = 0.520) and the number of words during the evening recall (t = −1.11, *p* = 0.271) (Table 4). Although the consolidation rate was not significantly different between STs and CNTs, we observed a trend indicating a worse memory performance (forgetting rate) in the STs group (t = −1.82, *p* = 0.078) (Table 4). Furthermore, there were no significant correlations between vocal activations and Gain (Table S4).

Starting with the results on the relationship between sleep variables and VBs, we carried out additional correlations to observe the potential relationship between performance and sleep in STs. Specifically, we correlated Gain and sleep features reflecting sleep fragmentation (ISW and SE). We revealed no significant differences (Table S4).

We also compared sleep variables on the eighth night between the two groups (Table S5) to ascertain that there were no differences in sleep variables (i.e., TBT, TST, SOL) that could have affected memory performance [23,24]. 

## 4. Discussion

The first aim of our study was to analyze the influence of ST on sleep and dream activity. Specifically, the retrospective questionnaire pointed to worse subjective sleep quality in STs than CNTs, while ST did not significantly affect daily sleepiness. A recent investigation into the quantitative characteristics of ST reported similar results, showing a lower sleep quality in subjects with highly frequent ST compared with subjects without other frequent parasomnias [25]. The longitudinal assessment of sleep variables found no significant differences between the two groups. The correlations, however, showed significant relations between VBs and sleep variables associated with sleep fragmentation, as measured by sleep diaries. 

Moreover, PSG information about ST indicated lower sleep efficiency than healthy subjects and altered macrostructure (i.e., lower % of REM and higher % of stage 2 NREM sleep) [2]. Therefore, VBs could reduce sleep continuity [2], negatively affecting the subjectively perceived sleep quality. A study on a larger sample would better clarify the relationship between ST and sleep.

Concerning oneiric activity, we found no significant differences between the two groups. However, correlations between dream variables and ST episodes showed interesting results. In particular, the negative correlations between the number of vocalizations (VBs) and emotional dream features might represent an intrinsic characteristic of dreaming in the ST population. Similarly, previous literature tried to detect specific dream features in other parasomnias, reporting that RBD’s dreams were intense and bizarre, while sleepwalking dreams were less immersive, complex, and bizarre [26,27,28]. Additionally, this evidence could also be explained by different sleep stages in which dream recall was collected (i.e., REM and N3). REM and NREM stages are characterized by a different kind of mental activity [29]. Specifically, REM was associated with more emotional, vivid, and bizarre dreams (“dream-like mentation”) than NREM, which was associated with reduced emotional intensity and more realistic oneiric content (“thought-like” mentation) [30,31,32,33]. ST can occur during all sleep stages but more frequently in the NREM phase [2,34,35]. This could explain the negative relationship between ST and emotional intensity. It is worth noting that the content of ST is often emotional, and there is a relationship between the VBs produced in REM sleep and the presence of an affective tone [1,34]. It is well-known that sleep mentation plays a crucial role in regulating emotions [36]. Dreaming attenuates the emotional intensity of the waking experience and promotes the assimilation of these salient emotional contents into existing memories [37,38]. ST episodes may help to overcome the well-known obstacles of exploring dream situations, favoring the understanding of the functioning mechanisms of this type of mental activity, which is not directly measurable [1]. In this vein, we suggest that the relation between VBs with low emotional intensity, specifically negative emotion, could reflect emotional regulation processes. However, information about sleep stages was missing in our study, and dream reports were collected in the morning after the last waking. Therefore, a study with multiple awakenings following ST episodes and the analysis of ST and oneiric contents might explain the relationship between ST and emotional intensity in relation to the EEG patterns preceding the dream recall [39,40].

The second aim focused on the relationship between sleep-dependent memory consolidation and VBs. We did not find a significant difference between STs and CNTs in memory performance and did not detect any correlation between VBs and overnight gain. The comparable number of trials and the number of correct words in the evening indicated appropriate learning in the STs group. Few studies investigate offline memory consolidation in these sleep disorders. It should be noted that our results show a trend toward increased forgetting in the STs group. Some authors suggested that not all sleep disorders were associated with impairment in memory consolidation (i.e., RBD or Somnambulism) [9,11]. Forgetting might occur when sleep was significantly disrupted [41] or depending on a greater post-learning level of arousal [42] (i.e., insomnia, obstructive sleep apnea, or narcolepsy [23]). Our qualitatively and prospectively assessed sleep variables did not present significant differences between the two groups. However, the recent PSG study by Mangiaruga et al. [2] revealed significantly lower sleep efficiency in ST. In this vein, ST may negatively affect sleep-dependent memory consolidation. A larger sample could provide substantial support for this result.

On the other hand, ST could be considered a model to investigate sleep-dependent memory processes. Uguccioni et al. [11] showed that ST could represent a replay of recently learned memories in specific circumstances. It is unclear if this “overt replay” reflects an improvement in the consolidation rate. The absence of objective polysomnographic measures associated with the reported assessment of sleep-dependent consolidation processes does not allow a clear conclusion on this issue.

### Limitations and Future Directions

ST is often related to other nocturnal behaviors [1,2], making the study of its peculiarities complex. Our research includes a selected group of sleep talkers without other nocturnal behavior disorders. However, the small sample size may have influenced some of the results, making it difficult to generalize these findings to the general ST population.

In this respect, we believe that considering a larger sample in a longitudinal design would allow the use of different models of statistical analysis (e.g., multilevel models) that may provide information about the temporal relationship between dependent and independent variables of interest [43].

Moreover, we did not include the audio recordings of the control subjects during the night. This may represent a limitation of our study since it did not allow us to assess the presence/absence of potential non-verbal sounds among control subjects.

A further limitation of this research is the absence of objective measurements (i.e., PSG). On the one hand, ST monitoring in an ecological environment enables the optimal investigation of the phenomenon of somniloquy. On the other hand, PSG recording allows us to identify the sleep stages where ST occurs and obtain quantitative information about the sleep of STs (micro- and macro-structure). ST episodes are often difficult to record with overnight video-EEG polysomnography in a sleep lab [44,45,46]. Therefore, future research could involve PSG home monitoring to study sleep in parasomnias.

Regarding the analyses on dream variables, the absence of PSG was a limitation in this study. As described above, the characteristics of dream recall are different among the stages of sleep [30,31,32,33], as well as the characteristics of ST episodes [4]. Moreover, dream report collection exclusively after the last awakening in the morning did not allow us to assess the oneiric content in relation to VBs. The literature about ST showed a high correspondence between dream content and sleep speech [1,6]. In this case, a PSG laboratory study with a protocol of multiple awakenings could provide evidence of EEG patterns of DEB and accurate analysis of dreams and VBs content among all sleep stages.

## 5. Conclusions

The ability to talk while asleep has always fascinated humans. To the best of our knowledge, this is the first study systematically investigating the qualitative features of sleep and dreams in ST and the influence of VBs on declarative memory consolidation. To summarize, our findings show that sleep talkers had worse subjectively perceived sleep quality when it was evaluated retrospectively. Consistently, VBs showed a significant relationship with sleep fragmentation. In a pioneering way, we provided the analysis of dream contents, revealing a negative relation between VBs and emotionality.

Concerning sleep-dependent memory consolidation, although we did not find a significant difference between the two groups, we observed a trend of forgetting in the STs group. On the one hand, the partial failure in the overnight gain could be due to specific sleep characteristics associated with STs. On the other hand, VBs could reflect processes of memory replay that would occur during sleep.

As previously discussed, dream processes modulate the consolidation of emotionally intense waking memories [36]. Therefore, what we observed through mental activity analysis could reflect the emotional regulation processes associated with waking elements.

Future research should consider the potential relationship between emotional processes and VBs. The literature reported that episodic waking material could be incorporated into the VBs [4,47]. Hence, ST may represent a model for investigating the overt replay of autobiographical material as much as semantic material.

Although our study was conducted in an ecological environment, some limitations should be mentioned, such as the small sample size and the lack of objective sleep measures. Therefore, the study should be replicated with a larger sample and PSG measures. Moreover, a multiple-awakening protocol to collect mental sleep activity immediately after VBs would help provide more information about ST as DEB and the cognitive processes associated with VBs [48].

Overall, we found support for the idea that ST could be an interesting phenomenon for investigating sleep-dependent cognitive processes, such as language processes, emotional regulation, and mental activity [1,2], allowing us to observe them as they occur.

## Figures and Tables

**Table 1:** Results of the comparisons concerning demographic characteristics.

Demographic Characteristics	STs = 29 Mean (sd)	CNTs = 30 Mean (sd)	Statistical Comparisons	*p*
Age	23.48 (2.81)	24.27 (3.24)	U = 366.000	0.291
Gender	F = 23 M = 6	F = 24 M = 6	χ^2^ = 0.004	0.948

Abbreviation: STs, Sleep Talkers’ group; CNTs, healthy subjects’ group; F, Female; M, Male.

**Table 2:** Comparisons results of retrospective and longitudinal sleep measures (sleep diaries).

**Retrospective Questionnaire**	**STs = 29** **Mean (sd)**	**CNTs = 30** **Mean (sd)**	**Statistical Comparisons**	**adj-*p***
PSQI	5.83(2.78)	3.90 (1.52)	U = 633.500	**0.032**
ESS	7.21 (3.84)	5.27(3.14)	t = −2.13	0.202
**Sleep Diaries Variables**	**STs = 29** **Mean (sd)**	**CNTs = 30** **Mean (sd)**	**Statistical Comparisons**	**adj-*p***
SOL	14.38 (10.14)	13.07 (6.02)	U = 420.000	0.937
ISW	4.16 (0.59)	5.67 (6.95)	U = 418.000	0.987
TBT	490.25 (53.31)	498.04 (63.57)	t = −0.509	1.000
TST	438.00 (39.86)	441.64 (41.33)	t = −0.344	1.000
SE (%)	89.86 (4.51)	89.33 (5.11)	t = 0.420	1.000
Sleep Depth	3.78 (0.56)	3.80 (0.49)	t = −0.102	0.919
Sleep Quiet	3.32 (0.64)	3.75 (0.59)	t = −2.669	0.080
Sleep Restless	3.40 (0.65)	3.56 (0.57)	t = −0.974	0.890

Significant results (*p* < 0.05) are highlighted in bold. Abbreviation: STs, Sleep Talkers’ group; CNTs, healthy subjects’ group; PSQI, Pittsburg Sleep Quality Index; ESS, Epworth Sleepiness Scale; SOL, Sleep Onset Latency; ISW, Intra-Sleep Wakefulness; TBT, Total Bed Time; TST, Total Sleep Time; SE, Sleep Efficiency.

**Table 3:** Comparison results of dream variables between STs and CNTs.

Dream Variables	STs = 28 Mean (sd)	CNTs = 24 Mean (sd)	Statistical Comparisons	adj-*p*
TWC	73.35 (61.89)	85.79 (65.47)	U = 296.000	1.000
EL	2.44 (1.11)	2.41 (0.81)	U = 326.000	0.910
VV	1.91 (0.88)	2.15 (0.84)	t = −1.017	1.000
B	2.99 (0.79)	3.29 (0.81)	t = −1.354	0.728
NW	0.66 (1.07)	0.44 (0.35)	U = 321.000	1.000
PW	1.38 (1.32)	1.32 (0.95)	U = 312.500	1.000

Abbreviations: STs, Sleep Talkers’ group; CNTs, healthy subjects’ group; TWC, Total Words Count; EL, Emotional Intensity; VV, Vividness; B, Bizarreness; NW, negative words; PW, positive words.

**Table 4:** Comparison results of WPT variables.

Word Pair Task Variables	STs = 28 Mean (sd)	CNTs = 30 Mean (sd)	*t*-Test (d.f.)	*p*
Gain	−0.14 (1.18)	0.53 (1.63)	t = −1.817 (56)	0.078
Evening Immediate Recall	40.04 (5.77)	41.70 (5.64)	t = −1.110 (56)	0.272
Numbers of trials	1.57 (0.57)	1.67 (0.55)	t = −0.648 (56)	0.520

Abbreviations: STs, Sleep Talkers’ group; CNTs, healthy subjects’ group.

## Data Availability

The data presented in this study are available on request to the corresponding author.

## Supplementary Materials

The following supporting information can be downloaded at: https://www.mdpi.com/article/10.3390/jcm11216489/s1, Table S1: Questionnaire assessing the general health involved in the Online-survey during the first step of the recruitment process; Table S2: Example of Dreams evaluation of ST subject performed by independent judges; Table S3: Correlation matrix of STs between Vocal Activation (VBs and NVBs) and sleep and dreams variables collected during the experimental week; Table S4: Correlation matrix of STs between Gain, Vocal Activation (VBs and NVBs), and sleep variables during the eighth night; Table S5: Comparison results of sleep variables during the eighth night.
