# Supplementary material for: The Influence of Sleep Talking on Nocturnal Sleep and Sleep-Dependent Cognitive Processes
Source: J Clin Med. 2022 Nov 1;11(21):6489. doi: 10.3390/jcm11216489 (PMC9658338; doi:10.3390/jcm11216489)
Supplement: Supplementary file 1 [file jcm-11-06489-s001.zip › jcm-1960113-supplementary.pdf]

Supplementary materials

Table S1. Questionnaire assessing the general health involved in the Online-survey during the first step recruitment process

Questionario che valuta la salute generale	Questionnaire assessing the general health
1. Sei destrimane, mancino o ambidestro? * a. Destrimane b. Mancino c. Ambidestro	1. Are you right-handed, left-handed or ambidextrous? * a. Right-handed b. Left-handed c. Ambidextrous
2. Hai problemi di salute acuti o cronici (problemi medici o psicologici)? ((Se sì specificare in "altro”) * a. Si b. No c. Altro	3. Do you have acute or chronic health problems (medical or psychological problems)? (If yes, please specify in "other”) * a. Yes b. No c. Other
3. In questo periodo stai assumendo qualche tipo di farmaco, inclusa aspirina, pillola anticoncezionale, antiasmatici, ecc... (n.b vale anche se fai uso di sostanze stupefacenti)? (Se sì specificare in "altro”) * a. Si b. No c. Altro	4. Are you taking any kind of medication currently, including aspirin, birth control pills, anti-asthmatic drugs, etc.? (n.b. also applies if you are using drugs)? (If yes please specify in "other”) * a. Yes b. No c. Other
4. Dormi da solo o condividi il letto/ la stanza con un'altra persona? * a. Da solo b. Con un’altra persona	5. Do you sleep alone or share your bed/room with another person? *. a. By yourself b. With another person

5. Ti risulta facile abituarti a dormire in ambienti del tutto nuovi? *<br><br>a. Si<br>b. No	6. Do you find it easy to get used to sleeping in completely new environments? *<br><br>a. Yes<br>b. No
6. Con che frequenza dormi durante il giorno (volte a settimana)? *	7. How often do you sleep during the day (times per week)? *
7. Specificare se la frequenza dei sonnellini pomeridiani dipende dalla presenza/assenza di bisogno di sonno o dalla possibilià/impossibilità di tempo libero per dormire (es. orari di lavoro, lezioni universitarie, ecc…) *	8. Specify whether the frequency of afternoon naps depends on the presence/absence of sleep needs or on the possibility/impossibility of free time to sleep (e.g., work schedule, university classes, etc...) *
9. Quanti sogni ricordi al risveglio in una settimana?	9. How many dreams do you remember waking up in a week?
10. Quanto sono vividi i pensieri e le immagini durante il sonno? Indica un numero tra 1 (per nulla) e 10 (molto vividi). *	10. How vivid are thoughts and images during sleep? Give a number between 1 (not at all) and 10 (very vivid). *

Table S2. Example of Dreams evaluation of ST subject performed by independent judges.

**Valutazione dei sogni**	**Dreams evaluation**
**Trascrizione:** Mi ricordo di aver fatto anche un altro sogno che era abbastanza terrificante ma non riesco a ricordarlo l'unico pezzo che ricordo forse è che a un certo punto di questo sogno ero con mia sorella e i miei cugini e dovevamo fare una gara a chi arrivava prima a casa dei miei cugini scendendo diciamo facendo una strada bel essenzialmente tutta una serie di vie in discesa e non so come ma io e mia sorella siamo arrivate prima giù e i miei cugini sono rimasti indietro qualcuno credo si sia anche fatto un po' male eee niente diciamo era un sogno un po' sogno non so perché ero così in competizione con i miei cugini per arrivare a casa di uno di loro per prime e appunto ricordo solo di aver fatto cose molto acrobatiche il che insomma era molto strano che io non avessi paura di fare determinati salti o cose del genere e poi l'ultima parte di questo sogno che riesco a ricordare perché c'è dell'altro era solo che ero in casa ed era pieno di animali det e alcuni non riuscivo a capire se erano pericolosi erano nascosti un po' ovunque in mezzo alla verdura c'era un serpente che non so come ho fatto io a vedere mia madre lo ha fatto uscire eee subito dopo c'era un'alt un felino molto piccolo era un cucciolo ma diciamo era faceva abbastanza male quando mordeva e non ricordo credo anche altri animali e devo dire che anche se lì per lì ero anche abbastanza spaventata la fine di questo pezzo è evoluta in maniera più positiva nel senso che in certo senso sono riuscita forse legare non legare con gli animali era come se non c'ave non fossero più aggressivi nei miei confronti a un certo punto non ricordo come abbia fatto io a domarli credo di essere anche riuscita fuori a cercare il serpente che avevamo cacciato perché mi sentivo in colpa ed era diventato un diciamo un gatto affettuoso	**Transcription:** I also remember having another dream that was quite terrifying but I can't remember it the only bit I remember maybe is that at one point in this dream I was with my sister and my cousins and we were supposed to have a race to see who could get to my cousins house first by going down let's say making a nice road essentially a whole series of downhill ways and I don't know how but me and my sister got down first and my cousins were left behind somebody I think even got a little hurt eee hmm nothing let's say it was a bit of a dream I don't know why I was so competing with my cousins to get to one of them's house first and just I just remember doing very acrobatic things which I mean it was very strange that I wasn't afraid to do certain jumps or things like that and then the last part of this dream that I can remember because there's of the other it was just I was in the house and it was full of det animals and some I couldn't tell if they were dangerous they were hidden a little bit everywhere in the middle of the vegetables there was a snake that I don't know how I got to see my mother got it out and right after that there was an oth- a very small feline it was a puppy but let's say it was hurt quite a bit when it bit and I don't remember I think other animals too and I have to say even though there and then I was also quite frightened the end of this piece evolved in a more positive way in the sense that in a way I managed hmm maybe to bond not bond with the animals it was like they had- they were no longer aggressive towards me at one point I don't remember how I tamed them I think I even managed to go out and find the snake that we chased away because I felt guilty and it had become a let's say an loving cat
**Pruning:** era abbastanza **terrificante** ero con mia sorella e i miei cugini e dovevamo fare una gara a chi arrivava prima a casa dei miei cugini facendo tutta una serie di vie in discesa e io e mia sorella siamo arrivate prima giù e i miei cugini sono rimasti indietro qualcuno si sia fatto un po' **male** aver fatto cose molto acrobatiche era molto strano che io non avessi paura di fare determinati salti e poi ero in casa ed era pieno di animali alcuni non riuscivo a capire se erano **pericolosi** erano nascosti un po' ovunque in mezzo alla verdura c'era un serpente mia	**Pruning**: it was pretty **terrifying** I was with my sister and my cousins and we had to have a race to see who could get to my cousins' house first by doing a whole series of downhill routes and my sister and I got down first and my cousins were left behind somebody got **hurt** a little bit having done very acrobatic things it was very strange that I wasn't afraid to do certain jumps and then I was in the house and it was full of animals some I couldn't tell if they were **dangerous** they were hidden a little bit everywhere in the middle of the vegetables there was a snake my

madre lo ha fatto uscire e subito dopo c'era un felino molto piccolo era un cucciolo ma faceva abbastanza **male** quando mordeva anche se lì per lì ero **abbastanza spaventata** è evoluta in maniera più positiva gli animali era come se non fossero più aggressivi nei miei confronti a un certo punto io essere anche riuscita fuori a cercare il serpente che avevamo cacciato perché mi sentivo in **colpa** ed era diventato un gatto **affettuoso**	mother got it out and next thing I knew there was a very small feline it was a puppy but it **hurt** quite a bit when it bit even though there and then I was **quite scared** it evolved in a more positive way the animals it was as if they were no longer aggressive towards me at one point I even managed to go outside and look for the snake we chased away because I felt **guilty** and it had become an **loving** cat
**Valutazione:**	**Scoring**:
**TWC:** 179	**TWC**: 179
**EL:** 5	**EL**: 5
**B:** 6	**B**: 6
**VV:** 5	**VV**: 5
**Pw:** 1	**PW**: 1
**Nw:** 8	**NW**: 8

Abbreviations: TWC, Total Words Count; EL, Emotional Intensity; VV, Vividness; B, Bizarreness; NW, negative words; PW, positive words.

Table S3. Correlation matrix of STs between Vocal Activation (VBs and NVBs) and sleep and dreams variables collected during the experimental week.

**Sleep Variables** N=29	**VBs** Coefficient Correlation *p*	**NVBs** Coefficient Correlation *p*
SOL	0.135+	-0.069+
	0.486	0.723
ISW	**0.420+**	0.212+
	**0.023**	0.279
TBT	0.071	0.104+
	0.716	0.591
TST	0.064	0.081+
	0.742	0.677
SE (%)	-0.011	-0224+
	0.953	0.242
Sleep Depth	**-0.441**	-0.071+
	**0.017**	0.714
Sleep Quiet	-0.052	-0.067+
	0.787	0.729
Sleep Restless	-0.214	-0.170
	0.265	0.377
**Dreams Variables** **N=28**		
**TWC**	-0.356+	-0.125+
	0.063	0.525
**EL**	**-0.416+**	-0.331+
	**0.028**	0.085
**B**	-0.173	-0.127+
	0.377	0.520
**VV**	-2.09	-0.088+
	0.286	0.657
**Pw**	-0.003+	-0.018+
	0.987	0.929
**Nw**	**-0.456+**	-0.215+
	**0.015**	0.273

Significant results (*p*<0.05) are highlighted in bold. Spearman's correlations are indicated with +

Abbreviations: STs, Sleep Talkers' group VBs, Verbal vocalizations ; NVBs, Non-verbal vocal activations ; TWC, Total Words Count; EL, Emotional Intensity; VV, Vividness; B, Bizarreness; NW, negative words; PW, positive words**.**

Table S4. Correlation matrix of STs between Gain, Vocal Activation (VBs and NVBs) and sleep variables during the eighth night.

Vocal Activation N=28	Memory Performance (Gain) Coefficient Correlation *p*
VBs	-0.050+
	0.806
NVBs	0.160+
	0.426
**Vocal Activation N=28**	
ISW	0.092+
	0.494
SE (%)	-0.134
	0.317+

Spearman's correlations are indicated with +

Abbreviations: STs, Sleep Talkers' group VBs, Verbal vocalizations ; NVBs, Non-verbal vocal activations; ISW, Intra-Sleep Wakefulness; SE, Sleep Efficiency.

Table S5. Comparisons results of sleep variables during the eighth night.

Eighth Sleep Diaries variables	STs=28 mean (sd)	CNTs=30 mean (sd)	Statistical comparisons	p
SOL	30.48 (25.96)	24.83 (21.13)	U= 456.00	0.566
ISW	5.89 (6.92)	16.37 (29.92)	U= 352.50	0.286
TBT	488.64 (14.18)	494.03 (15.11)	t= -1.399	0.167
TST	438.02 (33.59)	430.58 (38.87)	U= 457.00	0.564
SE (%)	89.89% (6.13)	87.27% (8.29)	U= 478.00	0.361

Abbreviations: STs, Sleep Talkers' group; CNTs, healthy subjects' group; SOL, Sleep Onset Latency; ISW, Intra-Sleep Wakefulness; TBT, Total Bed Time; TST, Total Sleep Time; SE, Sleep Efficiency.
